# Chasing Flies: The Use of Wingbeat Frequency as a Communication Cue in Calyptrate Flies (Diptera: Calyptratae)

**DOI:** 10.3390/insects13090822

**Published:** 2022-09-09

**Authors:** Julie Pinto, Paola A. Magni, R. Christopher O’Brien, Ian R. Dadour

**Affiliations:** 1 Discipline of Medical, Molecular & Forensic Sciences, Murdoch University, Murdoch, WA 6150, Australia; 2 King’s Centre, Murdoch University Singapore, Singapore 169662, Singapore; 3 Forensic Sciences Department, Henry C. Lee College of Criminal Justice and Forensic Sciences, University of New Haven, West Haven, CT 06516, USA; 4 Source Certain, Wangara DC, WA 6947, Australia

**Keywords:** Diptera communication, insect classification, *Lucilia sericata*, *Calliphora dubia*, *Sarcophaga crassipalpis*, *Musca vetustissima*

## Abstract

**Simple Summary:**

Some of the fastest and most agile flying insects are the calyptrate flies which include blow flies, flesh flies, and house flies. The current study compared the wingbeat frequency of four species, representing four genera of these flies, recorded in free flight and analyzed using sound editor and analysis software. Wingbeat frequency was found to differ between the four species and between sexes. Such findings provide insight into how flies are communicating and show promise for the use of wingbeat frequency as a novel methodology to identify fly specimens.

**Abstract:**

The incidental sound produced by the oscillation of insect wings during flight provides an opportunity for species identification. Calyptrate flies include some of the fastest and most agile flying insects, capable of rapid changes in direction and the fast pursuit of conspecifics. This flight pattern makes the continuous and close recording of their wingbeat frequency difficult and limited to confined specimens. Advances in sound editor and analysis software, however, have made it possible to isolate low amplitude sounds using noise reduction and pitch detection algorithms. To explore differences in wingbeat frequency between genera and sex, 40 specimens of three-day old *Sarcophaga crassipalpis*, *Lucilia sericata*, *Calliphora dubia*, and *Musca vetustissima* were individually recorded in free flight in a temperature-controlled room. Results showed significant differences in wingbeat frequency between the four species and intersexual differences for each species. Discriminant analysis classifying the three carrion flies resulted in 77.5% classified correctly overall, with the correct classification of 82.5% of *S*. *crassipalpis*, 60% of *C. dubia*, and 90% of *L. sericata*, when both mean wingbeat frequency and sex were included. Intersexual differences were further demonstrated by male flies showing significantly higher variability than females in three of the species. These observed intergeneric and intersexual differences in wingbeat frequency start the discussion on the use of the metric as a communication signal by this taxon. The success of the methodology demonstrated differences at the genus level and encourages the recording of additional species and the use of wingbeat frequency as an identification tool for these flies.

## 1. Introduction

The oscillation of insect wings during flight displaces air and produces a sound wave consisting of a series of harmonics, with the first harmonic referred to as the wingbeat or fundamental frequency [1]. The fundamental frequency of the flight sound is in direct correlation with the observed frequency of the wingbeat, with one cycle of flight sound corresponding to one cycle of wing motion [2,3]. Wingbeat frequency is a metric that has been explored for the identification of several insect species [4,5], with research dating back to the late-1800s [6,7,8,9,10,11,12,13]. Recently, there has been an increase in studies on wingbeat frequency as a potential surveillance and monitoring tool for insects associated with agricultural [14] and medical [15] importance, with the subject currently dominated by research on mosquitos [3,5,16,17], fruit flies [18], aphids [19,20], and butterflies [21]. Examining wingbeat frequency may also provide insight into insect communication, with flies potentially using the frequency as a signal to identify and communicate with conspecifics, especially if found to differ across species and sexes.

Wingbeat frequency is a principal kinematic feature of wing motion [22,23]. It varies considerably across insect orders, with most of the variability the result of body mass, wing loading, and wing area [24,25,26]. Wingbeat frequency ranges from the low 5–8 Hz frequencies of saturniid moths, to the high frequencies above 1000 Hz of small biting midges [27]. The evolutionary miniaturization of the insect body has decreased wingspan, necessitating an increase in wingbeat frequency along with other biomechanical adaptations to maintain ample aerodynamic force (lift) for flight [28,29]. Ample aerodynamic lift has been achieved by a high wingbeat frequency, enabled by the evolution of asynchronous flight muscles which contract as a series of oscillations from a single nerve action potential, and adaptations that enable the wings to rapidly rotate and change direction [29,30,31]. Such adaptations are evident in the complex flight mechanisms observed in Schizophoran flies which include some of the fastest and most proficient of the flying insects.

An observed interspecific and intersexual difference in wingbeat frequency alludes to the use of the characteristic in insect communication. As flies are considered non-eusocial, almost all communication used is in a sexual context [32] and the sex of the individual fly is, therefore, an expected and relevant variable when determining the function of wingbeat frequency. While differences in wingbeat frequency may fundamentally be incidental to flight, it is likely that these observed differences may be additionally subjected to evolutionary pressures. Wingbeat frequency is influenced not only by size, but also the sex and age of the individual, making such characteristics inherently linked to wingbeat frequency, and making wingbeat frequency a plausible secondary sexual trait. Wingbeats produce sound, but can also be perceived visually and aid in the dispersal of pheromones, suggesting it is a characteristic that is being received as a multimodal signal (intentional) or cue (unintentional) [33,34], complicated further by possible shifts in transmission [35]. The role of wingbeat frequency in calyptrate communication, as a signal or cue during courtship, far-field attraction, and during agonistic interactions such as male-male aggression, has yet to be resolved.

Calyptratae are a species-rich and highly diverse subsection of Schizophora containing the blow flies (Calliphoridae), house flies (Muscidae), and flesh flies (Sarcophagidae) [36]. This well-known clade exhibits a wide range of feeding strategies, including saprophagy, coprophagy, phytophagy, and parasitism, and exploits multiple substrates for breeding, including plants, mammal dung, carrion, necrotic tissues of living vertebrates, earthworms, and snails [37]. Calyptrates have often been used as subjects in flight mechanism research [38,39,40,41,42]; however, data on the “signature” wingbeat frequency of these flies is limited and has been collected under variable conditions using a range of methodologies that have often restricted free flight. The variability between published frequencies of the same species is likely to be the result of different experimental conditions, including the age of the recorded flies, the temperature, and flight restrictions placed on the flies (Table 1).

The use of acoustic microphones to record and classify the wingbeats of insects has largely been abandoned due to the low sound-to-noise ratio encountered [17,47], with few studies using the methodology since the 1990s [48,49]. While not recording wingbeats, the acoustic method has also been successfully used in the detection of hidden insect infestations in materials such as grain and wood [50]. The flight pattern of flies is one that is characteristically fast with rapid changes in direction, making the continuous close placement of a recording device during flight challenging. Studies in the past have tethered or otherwise confined flies to ensure proximity to the recording apparatus. It seems unlikely, however, that such methodology would produce data that can be generalized to natural conditions given that tethering has been shown to affect wingbeat frequency [51], it restricts flight behavior [39], and cage material creates acoustic interference [52]. Sound attenuates quickly even through air, with a reduction in the sound pressure level of 6 decibels when distance is doubled, a significant number given that the recorded subjects only produce around 30 decibels at 1 m. To obtain a continuous recording of a signal of ample intensity, the required methodology is one that enables recording proximal to a fast-moving subject and addresses noise.

Four calyptrate fly species were selected for this study: *Sarcophaga crassipalpis* Macquart, *Calliphora dubia* (Macquart)*, L. sericata,* and *Musca vetustissima* Walker, representing four genera: *Sarcophaga*, *Calliphora, Lucilia*, and *Musca*. All four species are commonly found in various habitats of Western Australia, including in bushland on decomposing remains, in paddocks on livestock dung, and in suburban backyards. *Sarcophaga crassipalpis, C. dubia,* and *L. sericata* are known carrion flies, frequently dominating carcasses in the collected area, including human (*S. crassipalpis* [53,54]; *C. dubia* [55]; *L. sericata* [56,57]), making the identification of these species important forensically.

*Sarcophaga crassipalpis* is a common, synanthropic species of flesh fly with a worldwide distribution that feeds on a variety of substrates. The species is a producer of facultative myiasis of humans, sheep, and reptilian hosts [58], and is ovoviviparous, enabling the depositing of live first instars directly onto carrion. *Sarcophaga crassipalpis* are often used as laboratory specimens in gene expression experiments and as a model for the study of aggression [59].

*Calliphora dubia*, also known as the blue-bodied blow fly, is distributed across southwestern and central–southern Australia [60]. The species is ovoviviparous [55], an early colonizer of carrion, responsible for primary facultative myiasis (IRD, pers. comm), and of agricultural interest as a pollinator [61].

*Lucilia sericata*, also known as the common green bottle fly, has a worldwide distribution with varying degrees of synanthropy, and uses a wide variety of food substrates for larvae development, including carrion as an early colonizer [62,63]. The species is the number one cause of primary facultative myiasis in sheep in the middle latitudes of Europe and is used in maggot therapy to treat necrotic wounds in humans [64]. The species was first recorded in Australia in 1911 when it was incorrectly implicated in sheep myiasis, until the correct species was identified as *L. cuprina* [65,66].

*Musca vetustissima* are predominantly nuisance flies attracted to the feces of animals and breeding in livestock dung [58]. *Musca vetustissima*, commonly referred to as the Australian bush fly, is found on all parts of the Australian mainland, Tasmania, southern Papua, and the larger inshore islands [67]. The species is a known nuisance fly, especially in intensive animal facilities such as cattle feedlots, and acts as a vector of infectious bovine kertoconjunctivitis and *Chlamydia trachomatis* [68]. As with all other recorded species, *M. vetustissima* avoids shaded areas unless very hot, and is found in a broad range of habitats including deserts, grazing lands, seashores, and suburban backyards. In the field, bush fly larvae develop primarily on animal feces and sometimes in the gut contents of carcasses [69].

The recording of these four species provides a case study for determining differences in the wingbeat frequency of species that are commonly found together, and the variability of wingbeat frequency between individuals of the same species and sex and over an individual recording. Recording flies individually also provides the opportunity to explore the sexual dimorphism of wingbeat frequency and its role in sexual communication. This research will provide insight into the pre-copulatory behavior of flies by exploring the use of wingbeat frequency as a far-field cue or signal. Whether quantifiable intergeneric and intersexual differences necessarily show that wingbeat frequency is a characteristic detectable and used by calyptrate flies will also be discussed in the context of the limits of fly morphology to perceive these cues either acoustically, visually, and/or in combination with chemical cues.

## 2. Materials and Methods

### 2.1. Fly Specimens

The wingbeat of 40 specimens (20 male and 20 female) for each of the four species (*S. crassipalpis, C. dubia, L. sericata,* and *M. vetustissima*) was recorded. Flies were sourced from field collections in Perth, Western Australia, and established in laboratory culture for approximately 12 to 15 generations. Adult flies were kept at 12:12 h light:dark cycle, 26 °C, with access *ad libitum* to water and granulated sugar. Flies were given weekly access to pet mince (beef and pork) both as a food source and as an oviposition medium. Once larvae were visible, the meat was removed and larvae were reared on additional pet mince on a substrate of dry sand in plastic rearing containers with fine mesh tops, at densities approximating 500. Pupae were removed from rearing containers and placed in plastic enclosures ~40 cm (L) × 20 cm (W) × 30 cm (H) with fine mesh tops and dry sand as a substrate. Emerged adults were given access to only water and granulated sugar. Specimens were not separated by sex. The day of emergence was designated day 0 and all flies were recorded at 3 (+/− 12 h) days. All flies were identified using published keys: Calliphorid [70], Sarcophagid [53], and Muscid [71].

### 2.2. Recording Protocol

Flies were recorded using a SONY ICD-PX470 voice recorder (50–20,000 Hz), using the recording mode LPCM 44.1 KHZ, with scene selection turned off. Recordings were saved and transferred as 16-bit PCM.wav files. The recording room was approximately 3 m × 3 m × 2.4 m (height), darkened with a ceiling light as the sole light source, and maintained at 26 °C. An hour prior to recording, a saturated towel was placed in the room to increase humidity. Immediately prior to releasing a fly specimen into the recording room, a 30 s sample of “noise” was recorded for each recording session. A single fly specimen was released into the room and recorded for approximately 2 min. The recorder was held extended and directed towards the fly maintaining a distance of ~1 m. If a specimen stopped flying, the recording was paused and the specimen was fanned to encourage flight.

Past research demonstrates that wingbeat frequency is affected by temperature [12,43], age [13], and the sex of the fly [13,44]. These variables were standardized in the current study by recording all specimens at the same age (3 days), temperature (26 °C), and the identification of the fly’s sex. During the first 48 h post emergence, flight ability is unpredictable and often unsustainable, with full flying ability not reached until day 3 [13]. Calyptrate flies emerge before flight muscles and associated enzymes are completely developed [27] and it takes several hours for haemolymph to be pumped into the wings, and up to 24 h for the body to be fully pigmented and expanded [72]. Three days post emergence is also the age of flies most encountered at traps [73], the age at which females are most attractive to male flies [44,74], and the age female flies mature eggs [75]. The optimal ambient temperature for insect activity varies according to species; however, most calyptrates tolerate or prefer temperatures from 20 to 30 degrees [76,77].

### 2.3. Analysis Protocol

Advances in sound editor and analysis software have made it possible to record and isolate low amplitude sounds in uninsulated environments. Unwanted noise can be reduced by using low and high-pass filters to eliminate sound below and above the expected frequencies and eliminate ambient noise such as tones at 50–60 Hz produced by domestic electricity. The use of spectral noise gating also reduces noise by applying noise reduction algorithms. Once noise is reduced, fundamental frequency detection (estimation) algorithms can be applied to isolate the signal. Blow flies share the frequency of their wingbeat with human speech, with 120 Hz the typical fundamental frequency for men and 210 Hz for women [78], making the use of speech analysis software such as Praat [79] suitable and useful.

Audio recordings were trimmed and cleaned using the audio editor Audacity^®^ Cross-Platform Sound Editor version 3.0.5 (https://www.audacityteam.org, accessed on 28 October 2021). The recording was reduced to a mono recording by splitting the obtained stereo recording into mono and deleting the second mono channel. The 30 s sample of ‘noise’ was used to create a profile for noise reduction using the default noise reduction settings (12 dB, sensitivity 6, frequency smoothing 3) and subtracted from the entire recording using the built in Fourier analysis as the noise reduction algorithm. The voiced introduction along with any overt noises such as the click of the recorder and accidental bumps were cut from the recording. Trimmed and cleaned recordings were saved (exported) as.wav files.

**Figure 1:**
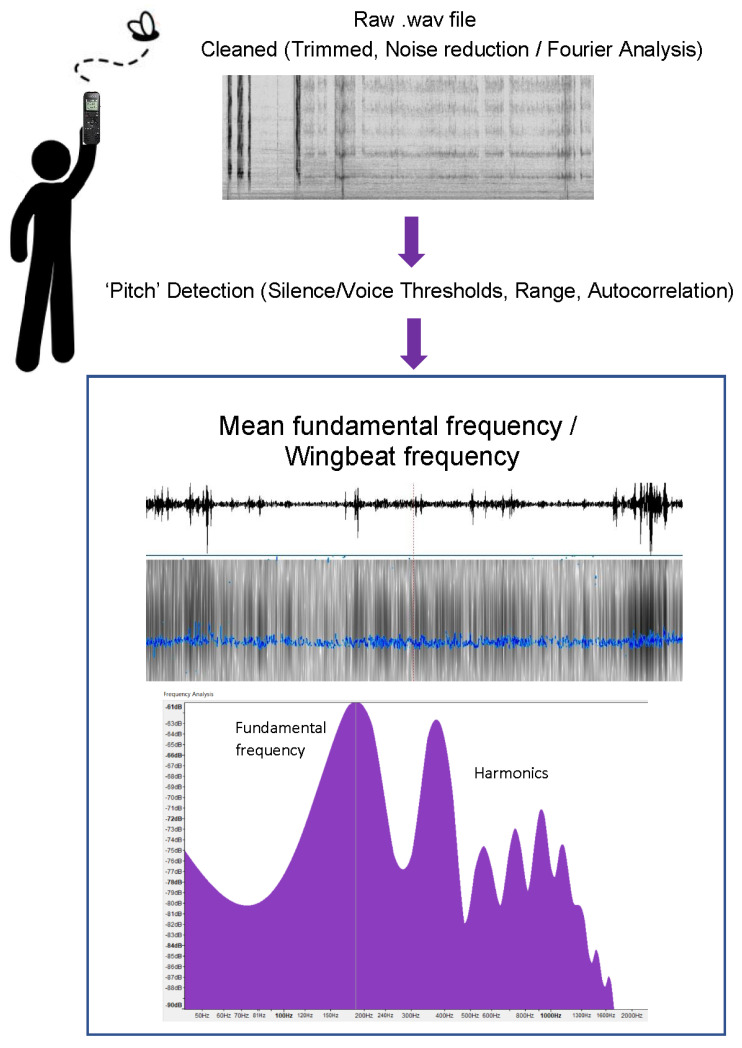
Overall methodology showing transition from raw recording to determination of mean wingbeat frequency for an individual specimen. Example shown is for male *C. dubia*.

Frequency analysis was completed on the trimmed recordings using the linguistic software Praat, version 6.1.55 (http://www.praat.org, accessed on 1 November 2021 [79] to obtain the mean wingbeat fundamental frequency for each individual recording. Pitch analysis settings were set to a Gaussian window: window length 6/75 (0.08 s), with a silence threshold of 0.01, a voicing threshold to 0.5, a voiced/unvoiced cost at 0.01, the pitch range set to 100 to 350 Hz, and autocorrelation was selected as the analysis method. The entire spectrogram was highlighted to obtain the mean fundamental frequency for each recording. Visible pitch contours were extracted for each individual recording and provided information on the number of valid (voiced) frames, estimated spreading at the 84% median, and range. Trimmed audio recordings of less than 1000 valid frames were discarded and not included in the analysis (Figure 1).

### 2.4. Statistical Analysis

IBM SPSS Statistics for Windows, version 28.0.0.1 (IBM Corp., Armonk, NY, USA) was used to perform all statistical analyses. One-way analysis of variance (ANOVA) was used to determine if differences are observed between the mean wingbeat frequencies of specimens of the same species and sex, and between overall variability (estimated spreading at the 84% median) between species and between sexes of a species. The effect size, eta squared (η^2^), was also calculated for each ANOVA test. Discriminant analysis was performed on the three carrion flies to classify by species, using the mean fundamental wingbeat frequency and sex as independents.

## 3. Results

All flies were recorded over a six-month period with *S. crassipalpis* recorded on eight separate days (six genetically different subgroups) in November, December, and February; *C. dubia* recorded on four separate days (four genetically different subgroups) in November and March; *L. sericata* recorded on five separate days (five genetically different subgroups) in November, January, and February; and *M*. *vetustissima* recorded on eight separate days (six genetically different subgroups) in December, April, and May.

A total of 672,821 wingbeat frequencies were collected across the four species (*S.* *crassipalpis* 230,968; *C. dubia* 206,533; *L. sericata* 132,150; *M*. *vetustissima* 103,170). The recording of valid signals varied across species and within species, with the two larger flies, *S. crassipalpis* and *C. dubia,* providing a higher number of signals than the two smaller flies.

**Figure 2:**
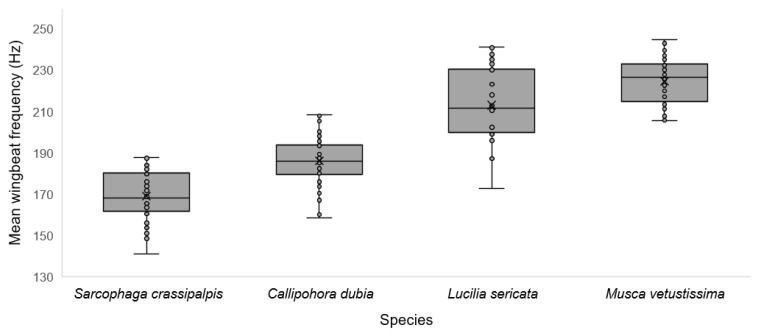
Mean wingbeat frequency of four recorded species. Mean is denoted with ‘X’ and upper and lower quartiles have been calculated using an inclusive median.

**Figure 3:**
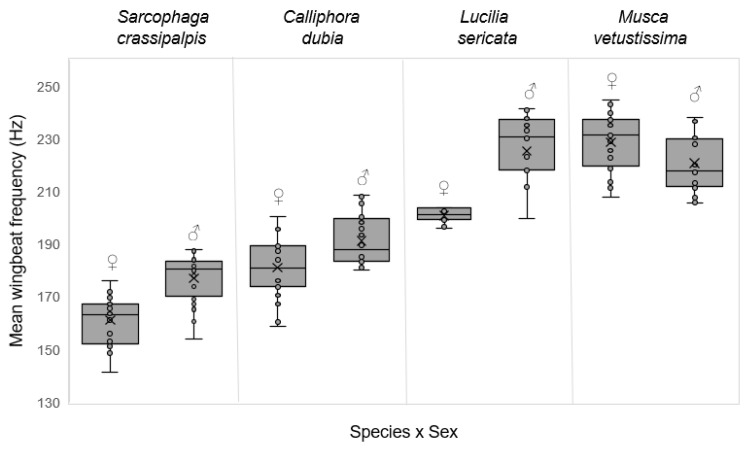
Mean wingbeat frequency of four recorded species separated by sex. Mean is denoted with ‘X’ and upper and lower quartiles have been calculated using an inclusive median.

The mean wingbeat frequency was significantly different between the four species [F_(3156)_ = 142.47, *p* < 0.001, η^2^ = 0.73], with the mean wingbeat for *S. crassipalpis* 169 Hz, *C. dubia* 186 Hz, *L. sericata* 213 Hz, and *M. vetustissima* 224 Hz. The mean wingbeat frequency differed significantly between males and females of each species: *S. crassipalpis* [F_(1,38)_ = 28.66, *p* < 0.001, η^2^ = 0.43]; *C. dubia* [F_(1,38)_ = 8.88, *p* = 0.005, η^2^ = 0.19]; *L. sericata* [F_(1,38)_ = 41.06, *p* < 0.001, η^2^ = 0.52]; and *M. vetustissima* [F_(1,38)_ = 5.37, *p* = 0.026, η^2^ = 0.12], with the mean wingbeat frequency of males significantly higher than females for all species except for *M. vetustissima* where the mean wingbeat frequency was significantly higher for female flies. The interquartile range was considerably less for *L. sericata* females compared to other species (Table 2, Figure 2 and Figure 3).

**Figure 4:**
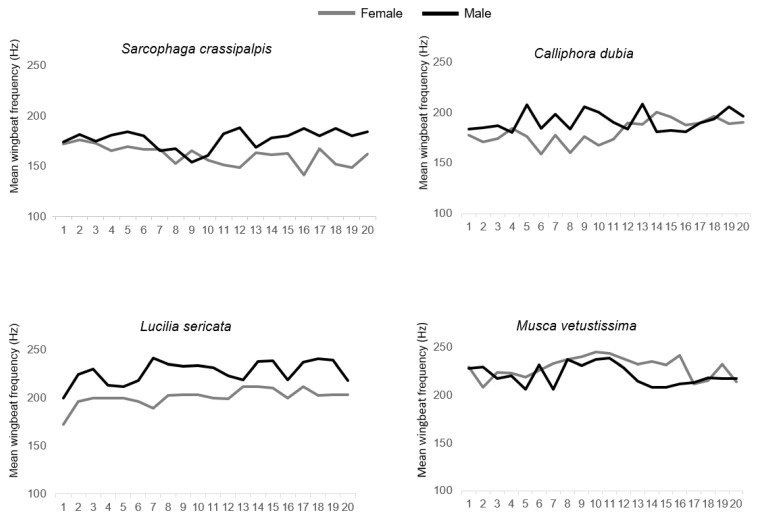
Individual specimen’s mean wingbeat frequencies for each of the four recorded species.

All flies exhibited relatively stable wingbeat frequencies over time, with the variability of individual fly recordings significantly different between the four species [F_(3156)_ = 28.42, *p* < 0.001, η^2^ = 0.35] and the average spread at 84% around the median 8.87 Hz for *S. crassipalpis,* 6.19 Hz for C. dubia, 11.36 Hz for *L. sericata*, and 10.32 Hz for *M. vetustissima*. Individual male flies showed significantly higher variability than females. Male flies showed significantly higher variability in *S. crassipalpis* [F_(1,38)_ = 9.57, *p* = 0.0037, η^2^ = 0.20] and *M. vetustissima* [F_(1,38)_ = 11.04, *p* = 0.002, η^2^ = 0.23], and marginally higher for *L. sericata* [F_(1,38)_ = 4.26, *p* = 0.046, η^2^ = 0.10] males. Variability did not differ between sexes of *C. dubia* (Table 2, Figure 4).

Discriminant analysis classifying the three carrion flies (*S. crassipalpis, C. dubia, and L. sericata*) resulted in 77.5% classified correctly overall, with 82.5% of *S. crassipalpis* correctly classified, 60% of *C. dubia* correctly classified, and 90% of *L. sericata* correctly classified, when both mean wingbeat frequency and sex were included as independents. Discriminant analysis using only wingbeat frequency resulted in a less accurate prediction model with 70.8% correctly classified overall, 67.5% of *S. crassipalpis* correctly classified, 62.5% of *C. dubia* correctly classified, and 82.5% of *L. sericata* correctly classified (Table 3).

## 4. Discussion

The significant differences between the wingbeat frequencies of the four recorded species suggests wingbeat frequency is being used by these flies as a communication cue or signal. The use of wingbeat frequency especially in regard to sexual communication is supported, with differences observed between the sexes of all four species. The inclusion of sex as an independent variable in the discriminant analysis resulted in a considerable increase in predictability in the classification model, further demonstrating the contribution of sex to the wingbeat frequency metric making it sexually dimorphic. Additionally, the observed difference in wingbeat frequency variability between sexes, with males showing increased variability compared to females (in three of the four species), also suggests that wingbeat frequency is more stable in females than males, and that female wingbeat may be a sexually selected characteristic being used by males to recognize suitable females.

The use of the autocorrelation algorithm utilized in the Praat software was key to successfully detecting the fundamental frequency of the recorded specimens. Testing of the algorithm used in Praat found the algorithm to be highly accurate, noise-resistant, and robust across frequencies in the range of the wingbeat frequencies and beyond [80]. The autocorrelation algorithm works in the lag (autocorrelation) domain, determining the similarity of signals as a function of the time lag between those signals. The fundamental frequency is then selected by considering the maximum of the autocorrelation function and the harmonics-to-noise ratio from the relative height of this maximum. The Gaussian window was used as it has been shown to produce better results than a Hanning window [80]. Use of the Praat software is widespread in the human literature [81,82], with some application in the insect literature [83,84]. Added parameters such as imposing a pitch floor and ceiling (with wide margins) around the previously cited fundamental frequency of calyptrate flies, and reducing the silence threshold below the default level, enabled the inclusion of the quiet insect sounds and enhanced the detection of the wingbeat’s fundamental frequency.

Why wingbeat frequency differs between the recorded species is a valid question and one that should take into consideration the observed difference between sexes. Body mass and wing size of the recorded specimens were not measured; however, such characteristics are observably different between the four species, and the difference in wingbeat frequency may be attributable to this difference in size. Body mass is a strong predictor of radiated acoustic power as the aerodynamic forces needed to stay aloft must be proportionally larger for heavier insects [85]. Past research demonstrates that variation in wingbeat frequency can be best described by incorporating body mass and wing area, of which the relative importance is 17.3% and 67.2%, respectively [26]. As larger wings produce more force per beat than smaller wings, fewer beats are needed per unit of time [26]. The overall structure, roughness of the wing surface, and wing vein topology also appear to contribute to wingbeat frequency [21].

Body mass and the linear dimensions of the wings may be fundamentally responsible for differences in wingbeat frequency, and such differences may have in turn been sexually selected. One example is frequency matching associated with the observed assortative mating of isolated molecular forms of morphologically identical *Anopheles gambiae* Giles [86], suggesting wingbeat frequency is not only dependent on morphology. Size differences are the result of numerous allometric traits, including life history, genetics, and physiology [5,87], and such differences can have important fitness consequences. Size can affect the ability of an organism to successfully occupy a habitat, succeed in predator prey interactions, and reproduce successfully [88].

Size may be a characteristic used by males when selecting a potential female, resulting in bigger females (also a slower wingbeat) dominating the population and providing a link between genotype and wingbeat. This trend is supported by the observed wingbeat frequencies of the four species in this study, with the mean wingbeat frequency decreasing as the size of the fly species increased. Sexual size dimorphism is common in the animal kingdom, with the adult size of a female often larger than the male, thought to be the result of the increased sensitivity of females to environmental conditions [89], and observed in many calyptrate flies including *L. sericata* [90]. A size difference may explain why the female mean wingbeat frequency was lower than the male of three out of the four recorded species.

Understanding how flies perceive and use wingbeat frequency is important to interpreting the results of this study. The more obvious modality to perceive this cue or signal is acoustically, and Diptera possess the morphology to perceive the recorded range of low frequencies [91,92,93]. Diptera antennae are capable of high mechanical sensitivity to sound stimuli, and their capacity to sense and use the mechanical vibrations of the antenna in response to airborne sound is well documented in mosquitoes, and in drosophilid and tephritid flies [93]. Diptera use the sound of wingbeat frequency both during the monitoring and control of flight, and in inter- and intra-specific communication. Antennal mechanics are involved in the control of flight maneuvers [1,42,94], with the mechanoreceptors on the antennae detecting changes in flight speed, as demonstrated in Drosophila [94,95] and calliphorid flies [93]. Flight control is often presented in the literature as the primary function of the female’s arista, with its role as a receptor of male courtship displays secondary, and antennal ‘hearing’ in flies as founded in the context of flight control and later evolving to include the additional function of acoustic communication [93,96].

Diptera audition is best suited to the detection of small stimuli in the near-field context [97]. Tachinidae and Sarcophagidae parasitoid fly species possess prothoracic tympanate ears and are able to eavesdrop on the far-field acoustic communications of cicadas and other orthopteran host species [98]; however, this is the exception in Diptera, with the vast majority possessing chordotonal organs, long identified as transducers of near-field sounds. Airborne sound is comprised of two energy components: particle velocity and sound pressure, with particle velocity degrading at a much higher rate than sound pressure as the distance from the source of the sound increases. Tympanal ears of cicadas are optimized to respond to the pressure component, enabling long-distance communication, while chordotonal organs are sensitive to particle velocity, limiting their sensitivity to near-field sounds [91]. In addition, it has been demonstrated in Drosophila that the antennae are particularly sensitive to the detection of small stimuli, as species-specific tuning is amplitude-dependent, relying on the active amplificatory mechanical feedback from the flies’ auditory neurons [97].

The acoustic use of wing beat frequency as a courtship signal has been documented in several Diptera families, notably Culicidae and Drosophilidae. The Culicidae family use the audible tone of wing movement to both recognize conspecific females in the pre-copulatory mode and during the subsequent courtship [86,97]. Male and female mosquitoes use wingbeat frequencies to recognize conspecifics and, depending on the species, they either modulate their wing-beat frequencies to converge at the same wingbeat frequency when the two wingbeat frequencies are similar [99], or they alter their wingbeat frequencies to share a higher harmonic when the wingbeat frequencies vary considerably and a shared frequency would be incompatible with flight [100,101,102,103]. Male and female mosquitoes also modulate their wingbeat frequencies when interacting with the same sex, by diverging and stabilizing at different wing-beat frequencies to avoid overlap [99]. Wingbeat frequency is also a key component of Drosophilidae courtship. Male Drosophilidae send a pattern of airborne vibrations with their wings known as courtship songs to female antennae, which include a sine hum, with a fundamental frequency between 100–350 Hz, and a pulse component. These songs differ in their spectrotemporal composition across species, especially regarding the interpulse interval during the pulse song, which is considered a crucial element for species recognition and a premating barrier [104,105,106,107,108]. Species-specific differences in courtship song structure are also evident in the receiver, with the antennae of species mechanically tuned to different frequencies and correlated with conspecific courtship songs [97].

Relatively less is known of the mating behavior of Calyptrates, which has historically been described as simple and swift after contact in the air or on a substrate. As more of the precopulatory and courtship behaviors of this clade are described, however, complex repertoires have been revealed [109]. As with Drosophila spp., calyptrate courtship includes an array of stereotyped behaviors such as orienting, tapping, waving, waggle, arching, and mounting [110,111], with wing vibrations by male flies a key component. Wing vibrations are commonly observed as a courtship behavior in several calyptrate genera, including calliphorids (*Protophormia terrae-novae* (Robinson-Desvoidy) [112]; *Chrysomya flavifrons* (Aldrich) [111]; *Lucilia cuprina* (Wiedemann) [74]), muscids (*M. domestica* and *Musca autumnalis* De Geer [113,114]), and sarcophagids ([115]; *Blaesoxipha stallengi* (Lahille) and *Sarcophaga ruficornis* (Fabricius) [116]). The quantification of courtship behaviors comparing investment and courtship success in the small hairy maggot blow fly, *Chrysomya varipes* (Macquart), demonstrated that there was no significant effect of wing vibration on mating success, strengthening the hypothesis that the role of this energy-expensive behavior is for mate recognition, as seen with the interpulse intervals of Drosophila courtship songs [110].

Whether the measured genus-specific and sexually dimorphic differences in wingbeat frequency of the recorded species are also reflected in the receivers of this signal, is key to understanding how wingbeat frequency is used. It has been shown in members of the *Drosophila melanogaster* Meigen species group that antennae of different species are mechanically tuned to different best frequencies, and these best frequencies correlate with high-frequency pulses of the conspecific courtship songs [97]. This conflicts with earlier work showing that with *D. melanogaster* the frequency of best antennal mechanical sensitivity (370 Hz) was considerably higher than the tones produced by wing vibrations during courtship [104,117]. Studies have also reported that the ablation of the arista and wing clipping reduces the male’s motivation to court, and results in males failing to attract the attention of nearby receptive females [93,104]. A key component of whether conspecific individuals are ‘listening’ to a signal depends on if the receiver’s antennae are operating in an active or passive mode around the best frequencies. It was determined that during flight, *D. melanogaster* antennae are not actively tuning into the wingbeat frequency (ranging from 145 Hz to 213 Hz) but rather passively tuned into a much higher range of 789 Hz to 991 Hz [97]. Alternatively, during conspecific courtship song, the receiver’s antennae operate in their active, non-linear mode and around the best frequencies of 147 Hz to 293 Hz, when courtship song emissions range from 127 Hz to 423 Hz [97]. Riabinina et al. (2011) suggest that the observed correlations between wingbeat frequency and the antennae best frequencies of *D. melanogaster* may be rather an indirect effect of the shared neuromuscular substrate for song production and flight rather than an adaptive co-tuning, as seen in mosquitoes. As the antennae use active mechanical feedback amplification that is sensitive to the detection of small stimuli, the antennae best frequencies of the receivers match those observed during relatively small stimuli such as conspecific song pulses, while larger stimuli such as wingbeat frequency during flight are tuned out [97]. It seems likely, therefore, that calyptrate males, like Drosophilidae, and unlike Culicidae, are recognizing conspecific females using visual and chemical far-field cues, in addition to (or rather than) audition, during the precopulatory stage.

Wingbeat frequency may be associated with chemical cues, as odor detection during flight is enhanced when pulsed at frequencies consistent with the wing beat [118]. The communicative role of volatile insect sex pheromones such as cuticular hydrocarbons (CHCs) has long been established in Diptera [119,120], and have been shown to be species-specific and sexually dimorphic in many calyptrate flies including calliphorids [74,121], sarcophaga [122], and Musca [119]. CHCs alone, however, are often not sufficient to elicit sexual behavior from either of the sexes, as demonstrated with *Ch. varipes* [123], and in Drosophila, where the identity of the sex is unknown until the male has touched and/or oriented toward the female [74,124,125]. While the odor of female flies does have a stimulatory effect on males, a greater effect is seen when the males are able to contact the females, suggesting that chemical cues are not being used as far-field cues, at least in isolation, and that visual cues may play a larger role.

Calyptrate eyes have evolved as part of an elaborate visual system to support their advanced flight abilities [126]. Calyptrates possess large eyes with highly dense areas of ommatidia, capable of high spatial resolution and detecting small, fast flying objects [126]. They also possess a high flicker fusion frequency which facilitates rapid temporal visual discrimination, enabling flies to perceive extremely fast or brief visual stimuli such as wing flashes [44]. The compound eyes of calyptrate flies are predominantly sexually dimorphic, with the eyes of males much larger than females, and in many species, including *M. domestica,* have male-specific photoreceptors (a lovespot) that respond more strongly and faster than female photoreceptors, translating to the improved resolution of small, fast-moving targets during pursuit [127]. During courtship, many calyptrate flies perform an ‘orienting” behavior. This behavior results in both flies facing each other after the male approaches a female and orients in a circular fashion to engage the female from the front. This behavior appears to constitute the first discretely sexual cue received by the female [111]. All recorded flies in the present study, excluding the flesh fly, *S. crassipalpis*, possess sexually dimorphic compound eyes.

Observations of male fly behavior during the precopulatory phase of mating supports the use of visual cues by males to recognize females during this phase. The males of many species of calyptrate flies routinely aggregate at visual markers such as hilltops (“hilltopping”) to wait for females to mate, or travel to feeding sites such as dung or carrion and perch on high vantage points such as twigs or leaves on the periphery to detect females [111,128]. Males of most dipteran groups, however, appear to locate conspecific females by trial and error, and attempt to copulate indiscriminately [32]. Examples include *Ch. flavifrons* males observed rapidly flying around carrion and approaching any object that approximates the size and shape of a female [111], *Cochliomyia hominivorax* (Coquerel) observed on established “waiting stations” from which they dart at any insect flying by [129], sarcophagidae males attempting to copulate irrespective of genus, species, or sex [115] [128], *M. domestica* observed striking at any object of appropriate size and color [113], and Drosophila males observed investigating any ambulatory individual that approximates the size of a female and comes within a few millimeters, and identification is only made by approaching and tapping the female [130]. Such behavior, however, may not be indiscriminate at all, but rather deliberate in the context of male-male competition. Cook [131] observed that experienced male *L. cuprina* secured significantly more matings than virgin males, and that experienced males gained an advantage not only from past successful matings, but also from intrasexual competition [131]. *Lucilia cuprina* males were found to direct several mating attempts at other males before attempting to mate with a virgin female, and male-male competition resulted in more successful copulations. Prior exposure to rival males has also been shown to increase copulation duration in *D. melanogaster* [132].

The strongest evidence suggesting that calyptrate flies are using far-field visual cues has been attained from research identifying the frequency of pulsed light as a key mate recognition cue in *L. sericata* [44] and wing interference patterns (WIPs) shown to be species-specific and sexually dimorphic [133]. The videorecording of *L. sericata* wing movements has demonstrated that wing movements produce a single, reflected light flash per wingbeat. When exposed to light flashes of varying frequencies, *L. sericata* male flies are significantly more attracted to the reflected light flashes corresponding to wing flash frequencies of 178 Hz, which are characteristic of young, conspecific females [44]. The low mating propensity of *L. sericata* on overcast days may further support the use of this visual cue as direct sunlight is needed to produce the wing reflections. WIPs have also been implicated as visual signals in mate recognition in calyptrate flies. WIPs are stable structural colors displayed on insect wings visible only against certain backgrounds and have been found to be species-specific and sexually dimorphic in a range of taxa including species of the *Chrysomya* genus [133]. WIPs have yet to be studied in the genera of the present study; however, given their visual capabilities and behavior, it seems likely that such a characteristic would also be used by *Sarcophaga, Calliphora,* and *Musca* as a far-field visual cue. The sex-specific adaptions of male compound eyes, and the use of female wing flashes and WIPS as visual cues, offer a possible explanation for the sexually dimorphic wingbeat frequencies of *L. sericata* and the other species recorded in this study.

Compared to other calyptrate flies, a moderate amount of knowledge has amassed on intraspecific communication for *L. sericata*. The species has been shown to respond to semiochemicals from flies of the same and different species feeding or ovipositing on a resource [134], with geographically disparate strains of this species found to differ in their responses to wool odor [135,136]. The species also possesses sexually dimorphic eyes and has been shown to respond to visual cues during the precopulatory phase of mating, demonstrated by the attraction of males to the light flashes of female wings as discussed above [44]. In the current study, female *L. sericata* showed considerably less of a range in wingbeat frequencies, with an interquartile range of only 4 Hz observed around the mean, while the interquartile range for the males was 19, and all other species ranged from 10 to 16 Hz. This similarity may be a further indication that wingbeat frequency is being sexually selected, and males are using female wingbeat frequency as a mate recognition cue. *Lucilia sericata* has also been shown to have significant dimorphism in the shape of the left and right wings between male and females flies, with shape varying considerably between individuals in a given population [88]. Female *L. sericata* have been shown to be on average 10% larger than males [90], and this approximates the percentage decrease in observed wingbeat frequency between the sexes. The results of this study further support the use of wingbeat frequency as a characteristic being used by males in intersexual communication. It seems likely that the larger size of female *L. sericata*, linked also to a lower wingbeat frequency, has been a sexually selected characteristic of this species, and is most likely being used as a visual cue by males during mating.

*Sarcophaga crassipalpis* do not appear to exhibit courtship behaviors, including female mate-attraction or rejection behaviors. Mating is a rapid and aggressive encounter, and female choice is severely diminished or absent. Females routinely exhibit “upside-down” behavior and are then immobilized by the male prior to mating [59]. In lab conditions, males of the species have been shown to distribute themselves uniformly and occupy spatially separated waiting stations. The males defend these spaces and will pursue when a female enters. How males recognize females is unknown; however, like *L. sericata*, they have been shown to have a clear preference for sunny spots, which may be related to the use of wing flashes, or simply an attraction to the heat produced. Unlike *L. sericata*, however, the eyes of the *Sarcophaga* genus are not sexually dimorphic, while the species does exhibit other observable sexual dimorphism such as a more hairy male [137]. The results of this study demonstrate that the mean wingbeat frequency is different between the sexes of this species, with the females showing less variability in wingbeat frequency than males. Further research is needed, however, to determine if this characteristic is being used by species despite their absence of sexually dimorphic eyes and lack of courtship behaviors.

Unlike the other recorded species, variability did not differ between the sexes of *C. dubia*, and the species showed the least amount of variability between individuals. No courtship or pre-copulatory behaviors have been described for this species, nor for flies of the same genus. The species has sexually dimorphic eyes, and while sexual size dimorphism has not been measured in this species, it has been demonstrated in the same genus, with females significantly larger than males. An assumed larger size of females may, therefore, account for the lower wingbeat frequency observed in female *C. dubia.*

*Musca vetustissima* possess observable sexually dimorphic characteristics. Female eyes are dichoptic, while males possess almost holoptic eyes. A difference in wing placement also varies by sex, with the wings of female flies at rest lying almost parallel to the body, whereas in the male they form at an acute angle [67]. The mating behavior of *M. vetustissima* closely resembles that of the face-fly *M. autumalis.* Mating behavior involves inflight or substrate “seizure” by the male, the female exhibiting avoidance behaviors, and copulation lasting 60–125 min. The presence of light and other males was found to significantly increase the number of mated flies [138]. *Musca vetustissima* is usually smaller than *M. domestica* [67], and was the smallest fly tested with the highest wingbeat frequency. As with other recorded species, wingbeat frequency differed significantly between sexes of *M. vetustissima*; however, it was the only species where that male wingbeat was lower than the female. Species within the *Musca* genus show size sexual dimorphism [139] with females possessing larger bodies and wings; therefore, it is surprising that males exhibited lower wingbeat frequency. The mean size of *M. vetustissima* has been shown to vary substantially across seasons [140], and as female size is more sensitive to environmental conditions [89], it is possible that the females recorded may have been smaller in size than the males. The methodology used for the attainment of specimens differed compared to the other three recorded species and, as a result larvae were exposed to a range of temperatures. Future research should compare *M. vetustissima* raised in a temperature-controlled laboratory for the entirety of their lifecycle to shed light on this anomaly of males exhibiting a higher wingbeat frequency.

The results of this research demonstrate the specificity (at least to the genus level) of wingbeat frequency and its potential use as a classifier in both field and laboratory fly populations. Ambient environmental factors, especially temperature, and biological factors, including the age and sex of the fly, are factors that influence wingbeat frequency, and were, therefore, controlled variables in the present study. For this metric to be used to its full potential in the field, such factors need to be taken into consideration in the same way temperature is considered when calculating the post-mortem interval of human remains using fly developmental data [141]. The recording of flies at varying age, sex, and temperature is needed to determine the extent these factors influence wingbeat frequency and to provide reference data. Additionally, as more species are added to comparative studies, the limits of the metric can be understood, with an expected increase in overlap between frequencies, especially with species of the same genus [4,5]. The use of additional classifying behaviors should also be explored to improve classification, as variables such as the circadian rhythm of flight activity, known to differ in flies [142], and geographic distribution, have been successfully added in the past to improve classification accuracy [47].

Current wingbeat research has focused on the use of optical sensors which exploit changes in light intensity when an insect flies between a laser light source and an array of phototransistors [47,143,144,145]. An optical rather than acoustic method has the advantage of excluding ambient noise and presents as more energy efficient [17]. Optical sensors, however, are difficult to use with calyptrate flies as the fly will tend to land on the sensor and walk through, failing to register a wingbeat. Design changes should enable the future use of optical sensors and serve as complimentary to the acoustical methodology used in the present study. The obstacles inherent to detection of the fundamental frequency of a small signal in a relatively noisy environment are now possible to overcome because of advances in sound analysis software. Recordings of *L. sericata* and *M. vetustissima* produced less valid signals overall, most likely due to the low amplitude of these two species. The faster wingbeat frequency of the two smaller flies also made the continuous placement of the recording device next to these species less than ideal and may indicate the limits of suitably sized insects using the described technique in a comparably noisy condition. While the two smaller flies, *L. sericata* and *M. vetustissima*, produced less valid wingbeat frequencies than the larger flies, the technique still enabled the extraction of over 100,000 fundamental frequencies over the 40 flies for the smallest species, *M. vetustissima.*

Wingbeat frequency is an unusual communication signal as it is directly linked to locomotion [103], sharing a neuromuscular substrate for wing oscillations observed during courtship and during flight [146]. Wingbeat frequency is also linked to the body mass and the linear dimensions of wings which also often differ between species and sexes. This makes it difficult to decern whether the observed differences in wingbeat frequency across species and sexes are simply incidental and sexual selection pressures are also incidental, or the observed differences are being used by calyptrate flies to recognize conspecifics and potential mates. The intergeneric and intersexual differences observed in the four recorded species suggest that wingbeat frequency is a characteristic that is being used by calyptrate flies. A review of the morphology related to the detection of wingbeat frequency, and their use of acoustical, visual, and chemical cues, suggests that wingbeat frequency is a characteristic that is perceivable by calyptrate flies, especially as a visual cue either in isolation or part of a multimodal communication system. In blood feeding Diptera there has been a general transition of precopulatory and courtship behavior. Auditory recognition, aerial swarms, and rapid copulation, as seen in Culicidae, has transitioned to a substrate-based system that relies on visual and chemical recognition, courtship rituals, and longer copulations, as witnessed in acalyptrates such as Drosophilidae and in calyptrates [147]. In the higher flies at least, wingbeat frequency may be, either additionally or solely, perceived chemically and visually rather than through audition. A review of the related literature on how Diptera use and receive communication signals, often involving wingbeat, demonstrates that wingbeat frequency is complex and multimodal when used by calyptrate flies, and something that requires more research at the species level.

## Figures and Tables

**Table 1:** Published wingbeat frequencies of calyptrate flies collected under various conditions.

Species	Family	Wingbeat Frequency (Mean Hz)	Method	Reference
*M. domestica*	Muscidae	130	free flight in box	[10]
*M. domestica*	Muscidae	160–162	tethered, stroboscope	[13]
*M. domestica*	Muscidae	180	free flight in box, optical tachometer	[43]
*L. sericata*	Calliphoridae	190	tethered, microphone	[1]
*L. sericata*	Calliphoridae	178 (females) 212 (males)	free flight in box, video-recorded reflected light flashes	[44]
*P. regina*	Calliphoridae	~150	oscillograph	[45]
*L. caesar*	Calliphoridae	205	free flight in box, high speed camera	[46]
*C. vicina*	Calliphoridae	145	tethered, microtomographic imaging	[42]
*C. vicina*	Calliphoridae	158	free flight in box, high-speed camera	[39]
*C. vicina*	Calliphoridae	162	free flight in box	[10]
*C. vomitora*	Calliphoridae	215	free flight in box, high speed camera	[26]
*Sarcophaga* spp.	Sarcophagidae	150	free flight in box, high speed camera	[26]
*S. carnaria*	Sarcophagidae	200	free flight in box, high speed camera	[46]

*Musca domestica* Linnaeus, *Lucilia sericata* (Meigen), *Phormia regina* (Meigen), *Lucilia caesar* Linnaeus, *Calliphora vicina* Robineau-Desvoidy, *Calliphora vomitora* (Linnaeus), *Sarcophagia carnia* (Linnaeus).

**Table 2:** Mean wingbeat fundamental frequencies and variability.

Species		*Sarcophaga crassipalpis*	*Calliphora dubia*	*Lucilia* *sericata*	*Musca* *vetustissima*	*p*-Value
**Specimens recorded**		40 20 females 20 males	40 20 females 20 males	40 20 females 20 males	40 20 females 20 males	-
**Fundamental frequencies** **obtained**	Total	230,968	206,533	132,150	103,170	-
	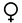	122,207	112,520	68,212	59,196	-
	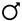	108,761	94,013	63,938	43,974	-
**Mean** **wingbeat** frequency (Hz) *	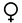 + 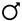	169 (18)	186 (14)	213 (30)	224 (18)	<0.001
	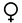	161 (14)	181 (15)	201 (4)	229 (16)	<0.01
	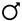	177 (10)	191 (15)	225 (19)	221 (15)	
**Mean** variability **	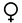 + 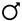	8.87	6.19	11.36	10.32	<0.001
	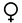	7.82	6.21	10.39	8.74	<0.05 (except *C. dubia*)
	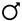	9.91	6.17	12.33	11.90	

* interquartile range shown in (·). ** average spread at 84% around the median (Hz).

**Table 3:** Discriminant analysis of the three carrion flies using mean wingbeat frequency and sex as independents.

Predicted Group Membership		Classification Results *			
		1 *Sarcophaga crassipalpis*	2 *Calliphora dubia*	3 *Lucilia sericata*	Total
Original Count	1	33	7	0	40
	2	12	24	4	40
	3	0	4	36	40
%	1	82.5	17.5	0	100
	2	30	60	10	100
	3	0	10	90	100

* 77.5% of original groups cases correctly classified.

## Data Availability

The data presented in this study are available on request from the corresponding author.
